# Endoscopic Ultrasound‐guided Fine‐Needle Biopsy With End‐Cutting Needles in Autoimmune Pancreatitis: A Systematic Review and Meta‐Analysis

**DOI:** 10.1002/deo2.70239

**Published:** 2025-11-04

**Authors:** Antonio Facciorusso, Maria Cristina Conti Bellocchi, Nicolò De Pretis, Luca Frulloni, Stefano Francesco Crinò

**Affiliations:** ^1^ Department of Experimental Medicine Università del Salento Lecce Italy; ^2^ Department of Medicine Gastroenterology and Digestive Endoscopy University of Verona Verona Italy

**Keywords:** acute pancreatitis, EUS‐FNA, EUS‐FNB, IgG4, pancreatic cancer

## Abstract

**Objectives:**

There is limited evidence on the diagnostic yield of endoscopic ultrasound (EUS)‐guided tissue acquisition (TA) using fine‐needle biopsy (FNB) in autoimmune pancreatitis (AIP), particularly considering the newer end‐cutting needles. The aim of this meta‐analysis was to provide a pooled estimate of the diagnostic performance of EUS‐TA using FNB in AIP patients according to the needle type used.

**Methods:**

A computerized bibliographic search was performed through April 2024. Pooled effects were calculated using a random‐effects model. The primary endpoint was diagnostic accuracy. Secondary outcomes were sample adequacy, rates of adequate material for levels 1 and 2 of histological diagnosis, definitive diagnosis reached with histology in addition to imaging/laboratory tests, and safety.

**Results:**

Twelve studies (three prospective series and one randomized trial) with 496 patients were included. Overall diagnostic accuracy rate was 75% (66%–83%), with a superiority of end‐cutting needles over reverse bevel needles (80%, 70%–90% versus 49%, 21%–67%; *p* < 0.001). Franseen (81%, 68%–93%) and Fork‐tip needles (86%, 74%–98%) showed the highest accuracy. Sample adequacy rate was 92% (87%–98%), and EUS‐TA using FNB provided level 1 of histological diagnosis in 47% of cases (38%–57%) and level 2 in 23% (16%–30%). EUS‐TA using FNB provided a definitive diagnosis in 77% (63%–91%) of cases. Pooled rate of adverse event was 2% (1%–3%), mainly mild pancreatitis.

**Conclusions:**

End‐cutting needles showed high diagnostic yield in patients with AIP, with a low rate of adverse events, and should be preferred over reverse bevel needles.

## 1 Introduction

Autoimmune pancreatitis (AIP) is a fibroinflammatory disorder of the pancreatic parenchyma due to an autoimmune process. The International Consensus Diagnostic Criteria (ICDC) identifies two kinds of AIP with distinct histological features but similar imaging and clinical appearance: type 1 and type 2 AIP [1]. Type 1 is included in the spectrum of IgG4‐related disease [2], whereas type 2 may be associated with ulcerative colitis and is histologically characterized by granulocytic epithelial lesions [1, 2].

Focal forms of AIP may represent a difficult clinical challenge because imaging appearance may be confused with pancreatic cancer, hence the frequent need for endoscopic ultrasound (EUS)‐guided tissue acquisition (TA) to rule out malignancy and to provide a definitive diagnosis [3].

Since the aforementioned histological characteristics defining the histological diagnosis of AIP can be assessed only on core biopsy tissue samples, EUS‐TA's role in the diagnostic algorithm of these conditions was questioned in the past because of the difficulty of EUS‐TA using fine‐needle aspiration (FNA) in obtaining adequate specimens for histopathologic analysis [3].

The development of EUS‐TA using fine‐needle biopsy (FNB) needles has generated a great deal of interest in the field based on proposed advantages over EUS‐TA using FNA needles of improving procurement of samples with preserved tissue architecture and allowing for immunohistochemistry or special stains required for certain diagnoses, including pancreatic cancer and AIP [4].

Despite these theoretical advantages of EUS‐TA using FNB, old‐generation reverse bevel needles have not significantly increased the diagnostic yield as compared to EUS‐TA using FNA [5]; on the other hand, newer devices with end‐cutting design were found to significantly improve the diagnostic yield in patients with pancreatic masses and other solid lesions [6].

Although FNB needles are supposed to improve tissue capture, thus increasing the histological accuracy, there is limited evidence on the diagnostic performance of EUS‐TA using FNB in AIP; a previous meta‐analysis published by our group found a suboptimal diagnostic accuracy as high as 63% with EUS‐TA using FNB, although significantly superior as compared to EUS‐TA using FNA [7].

However, the meta‐analysis mentioned above included mainly studies with reverse bevel FNB as only a few reports using end‐cutting needles were available at that time [7]; hence, there is a need to systematically assess the increasing body of evidence in the field to better define its role in the diagnostic algorithm of patients with AIP in light of the recent studies with the end‐cutting needles.

The aim of this meta‐analysis is to provide an updated pooled estimate of the diagnostic performance and safety profile of all the available needles for EUS‐TA using FNB in patients with AIP.

## 2 Methods

### 2.1 Selection Criteria

Articles included in this meta‐analysis were randomized‐controlled trials (RCTs) or observational studies meeting the following inclusion criteria: 1) articles recruiting >10 patients with AIP undergoing EUS‐TA using FNB; 2) studies published in English; 3) articles reporting at least the primary outcome (diagnostic accuracy). Case reports, non‐endoscopic studies, review articles, and studies with EUS‐TA using FNA were excluded.

### 2.2 Search Strategy

**FIGURE 1:**
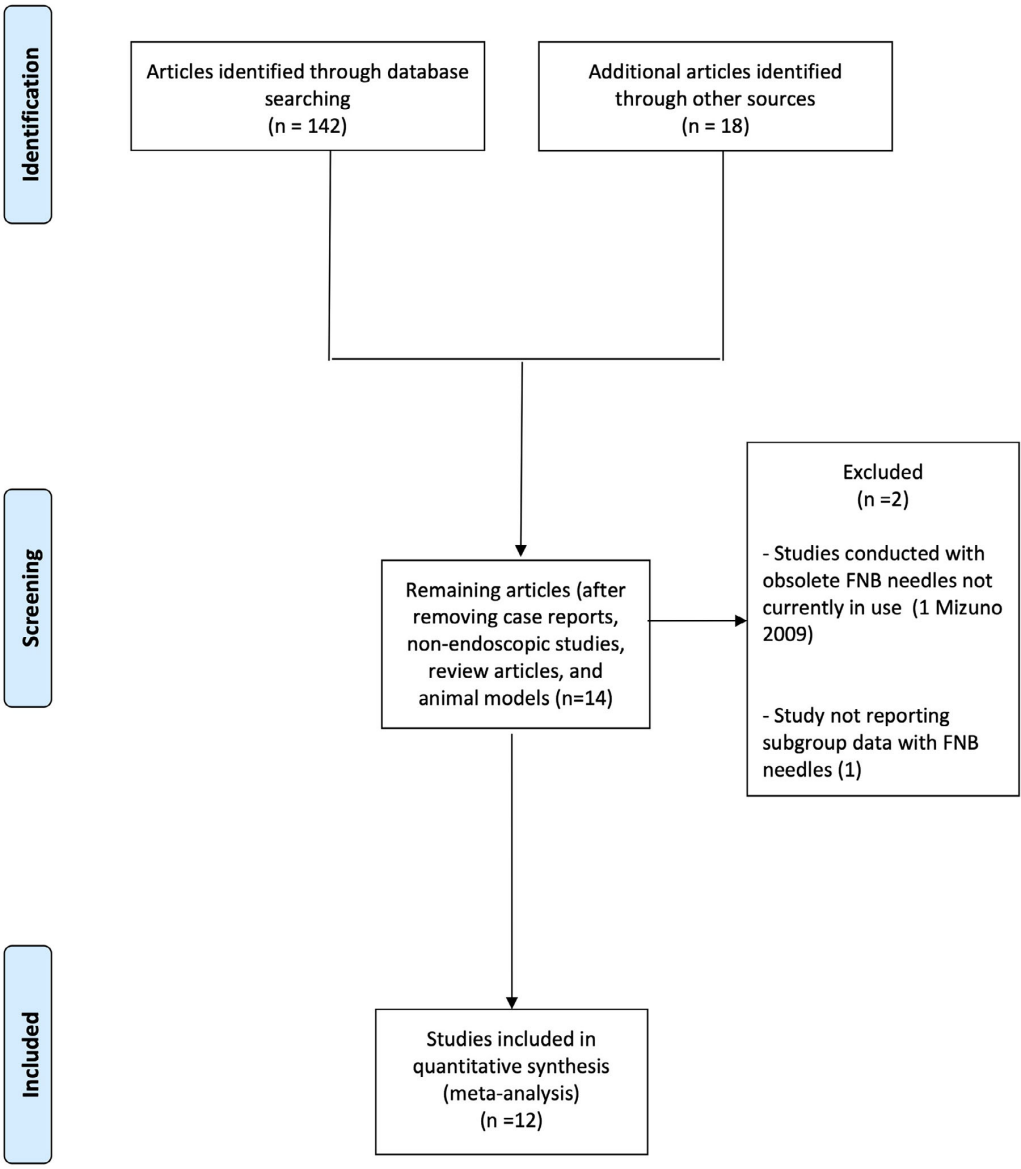
Flow chart of included studies.

Figure 1 reports the search strategy followed in the meta‐analysis.

Bibliographic research was conducted on PubMed, EMBASE, Cochrane Library, and Google Scholar, including all studies fulfilling inclusion criteria published until April 2024. The search string used in our meta‐analysis was: ((autoimmune pancreatitis) AND (endoscopic ultrasound [MeSH Terms])) OR (EUS [MeSH Terms]).

Relevant reviews and meta‐analyses on the use of EUS‐TA in patients with AIP were examined for additional eligible studies. Authors of included studies were contacted to obtain full text or further information when needed. Data extraction was conducted by two reviewers (Antonio Facciorusso and Stefano Francesco Crinò), and the quality of included studies was assessed by two authors independently (Antonio Facciorusso and Stefano Francesco Crinò) according to the Cochrane Collaboration's tool for assessing the risk of bias [8] for RCTs and the Newcastle‐Ottawa scale [9] for non‐randomized studies. Disagreements were solved by discussion and following a third opinion (Luca Frulloni).

### 2.3 Outcomes

The primary outcome was diagnostic accuracy, defined as true positive + true negative/ total number of patients, where true positive was considered the presence in the tissue sample of characteristic pathological features of AIP according to ICDC criteria (ICDC level 1 or 2).

Secondary outcomes were:

‐ sample adequacy (defined as the ability to procure histological samples adequate for interpretation).
‐ rates of adequacy for assessment of ICDC level 1 (at least 3 of the following: lymphoplasmacytic infiltration, storiform fibrosis, obliterative phlebitis, and abundant IgG4‐positive staining cells [defined as >10 positive cells per high‐power field]) [1] and ICDC level 2 of histological diagnosis of AIP‐1 (presence of 2 of the above‐reported features) [1].
‐ definitive diagnosis reached with histology (defined as the contribution of histologic findings obtained with EUS‐TA using FNB to the definitive diagnosis of AIP according to the ICDC when the diagnosis was not possible clinically based on imaging, serology, or other organs' involvement).
‐ adverse event rate.

### 2.4 Statistical Analysis

Diagnostic outcomes were pooled overall, then a sensitivity analysis based on the FNB needle used (whether end‐cutting or reverse bevel) was conducted. For the primary outcome (diagnostic accuracy), multiple sensitivity analyses were performed based on geographic location (East vs. West), specific end‐cutting needle used (Franseen vs. Fork‐tip vs. forward bevel), and restricted to AIP‐1 patients.

The meta‐analysis was conducted through a random‐effects model based on the DerSimonian and Laird test, and summary estimates were expressed in terms of rates and 95% confidence interval (CI). The pooled diagnostic outcomes of the different needles were compared using the bivariate approach [10].

Chi‐square and I^2^ tests were used for across‐studies comparison of the percentage of variability attributable to heterogeneity beyond chance. *p* < 0.10 for chi‐square test and I^2^<20% were interpreted as low‐level heterogeneity. The probability of publication bias was assessed using funnel plots.

All statistical analyses were conducted using Jamovi software [11]. A two‐tailed p‐value of less than 0.05 was considered statistically significant for all calculations.

## 3 Results

### 3.1 Characteristics of Included Studies

As shown in Figure 1, out of 160 studies initially identified, after preliminary exclusion of manuscripts not fulfilling inclusion criteria, 14 potentially relevant articles were examined. Among these studies, one was excluded as it used an obsolete needle that is not currently available [12], and another study was excluded because it did not report subgroup data with FNB needles [13]. Finally, 12 studies [14, 15, 16, 17, 18, 19, 20, 21, 22, 23, 24, 25] with 496 patients were included in the meta‐analysis.

The main characteristics of the included studies are reported in Table 1.

**TABLE 1:** Characteristics of included studies.

Study	Needle	Sample size	Study period/ Design/Diagnostic criteria	Country	Age	Gender male	IgG4 level (mg/dL)	Autoimmune Pancreatitis type 1	Mean number of needle passes	Range of pancreatic enlargement (diffuse)/ Use of ROSE
Jung 2015^14,b^	19G QuickCore in 28 pts 19G FNA in 3 pts 22G FNA in 10 pts 25G FNA in 1 pt 22G reverse bevel in 20 pts	62	2007–2013/ Retrospective/ICDC criteria	Korea	54.7 ± 15.3	49 (79%)	>135: 22 (35.5%)	NR	3.1	NR/No
Kurita 2020^15^	22G Franseen 20G forward bevel	50 51	2017–2018/ RCT/ ICDC criteria	Japan	70 (21–86) 68 (21–86)	40 (73%) 39 (71%)	406 (21.5–3440) 256 (12.9–3700)	100% 100%	NR	32 (58%)/No 28 (51%)/ No
Lee 2017^16,a^	19 G FNB 22G reverse bevel	15 42	2012–2015/ Retrospective/NR	Korea	NR	NR	NR	Type 1: 45 (78.9%) Type 2: 6 (10.5%)	2 (1–2) 3 (3–3.3)	NR/ No
Tsutsumi 2021^17^	21G Sonopsy CY	14	2015–2018/ Retrospective/ICDC criteria	Japan	71 (50‐79)	11 (78.5%)	364.3 (104–1090)	Type 1: 100%	4.5 (2–6)	10 (71%)/ No
Zator 2018^18,a^	19G, 22G, or 25G Acquire SharkCore ProCore	29	2012–2017/ Retrospective/ICDC criteria	USA	54	21 (72%)	>96: 7 (24.1%)	NR	2 (1‐7)	NR/ NR
Ishikawa 2020^19^	22G Franseen	56	2017/2019 Prospective/ ICDC criteria	Japan	66.7 ± 11.6	41 (73.2%)	447.9 ± 424.4	51 (91%)	2.2 ± 0.56	NR/ No
Oppong 2020^20^	Fork‐tip Reverse bevel	18 6	2011–2018 Retrospective/ ICDC criteria	UK	62.2 ± 11.4	17 (71%)	16 (70%) above normal limits	100%	2.55 ± 0.7 2 ± 0.7	NR/ No
Noguchi 2020^21,c^	22G Franseen 20G forward bevel	32	2013–2019 Retrospective/ NR	Japan	68 (17–83)	56.6%	62.3% above normal limits	100%	NR	NR/ No
Thomsen 2022^22,d^	22G Fork‐tip	19	2015–2020 Retrospective/ ICDC criteria	Denmark	NR	11 (57.8%)	NR	10 (52.6%)	2.5 ± 0.6	NR/ No
Notohara 2020^23^	22G Franseen 20G forward bevel/19G or 22G end‐cutting	27 58	NR Retrospective/ICDC criteria	Japan	65 ± 9	69 (81.1%)	>135: 75 (88.2%)	NR	3 (1–14)	28 (32.9%)/ No
Ishikawa 2024^24,e^	19G Franseen	20	2020–2023 Prospective/ICDC criteria	Japan	68 (60–71)	12 (60%)	277 (99–466)	100%	2 (2–2)	10 (50%)/ No
Conti Bellocchi 2023^25^	22G Franseen 22G Fork‐tip	7 32	2020–2022 Prospective/ICDC criteria	Italy	56.6 ± 18.3	31 (79.5%)	302 (58–1640)	26 (66.6%)	2 (2–2)	2 (5.1%)/ No

Data are reported as absolute numbers (percentages) or mean (± standard deviation or with interquartile range).

In the case of studies reporting different etiologies of pancreatic disease, only patients with autoimmune pancreatitis were considered.

^a^Study published only as a conference abstract.

^b^Only cases sampled with a reverse bevel needle were included in the analysis.

^c^Only cases sampled with FNB were included in the analysis and reported in the table.

^d^Only autoimmune pancreatitis patients were included in the analysis and reported in the table.

^e^Only cases sampled with a 19G Franseen needle were included in the analysis and reported in the table.

Abbreviations: ICDC, International Consensus Diagnostic Criteria; NR, Not Reported; RCT, Randomized Controlled Trial; ROSE, Rapid On‐Site Evaluation.

The recruitment period ranged from 2007 to 2023. One study was an RCT [15], and three were prospective [19, 24, 25]. Two studies were published as conference abstracts [16, 18]. Three studies also included patients who underwent EUS‐TA using FNA or non‐AIP patients [14, 21, 22], and one study [24] also included a historical cohort of patients overlapping with a previous publication from the same group; in these cases, only patients fulfilling the inclusion criteria of the meta‐analysis were included in our systematic review.

Three studies used first‐generation FNB needles (mainly reverse bevel) [14, 16, 17], and one study included both reverse bevel and end‐cutting needles without differentiation in the analysis [18]. The study by Oppong et al. [20] included mainly Fork‐tip needles with a small proportion of cases with reverse bevel FNB, the RCT by Kurita et al. compared 22G Franseen vs. 20G forward bevel needles [15], and the same needles were also used in two non‐randomized studies [21, 23]. The other studies used Franseen and/or Fork‐tip FNB needles.

Most included studies were conducted in Asia, whereas four were conducted in Western countries [18, 20, 22, 25].

The mean number of needle passes ranged between 2 and 3, and none of the included studies used the rapid on‐site pathological evaluation. The vast majority of studies included patients with type‐1 AIP.

Quality was deemed mainly high, with four retrospective studies assessed as low‐quality articles [14, 16, 18, 23]. Details on methodological characteristics and quality of included articles are shown in Table S1.

### 3.2 Diagnostic Accuracy

**FIGURE 2:**
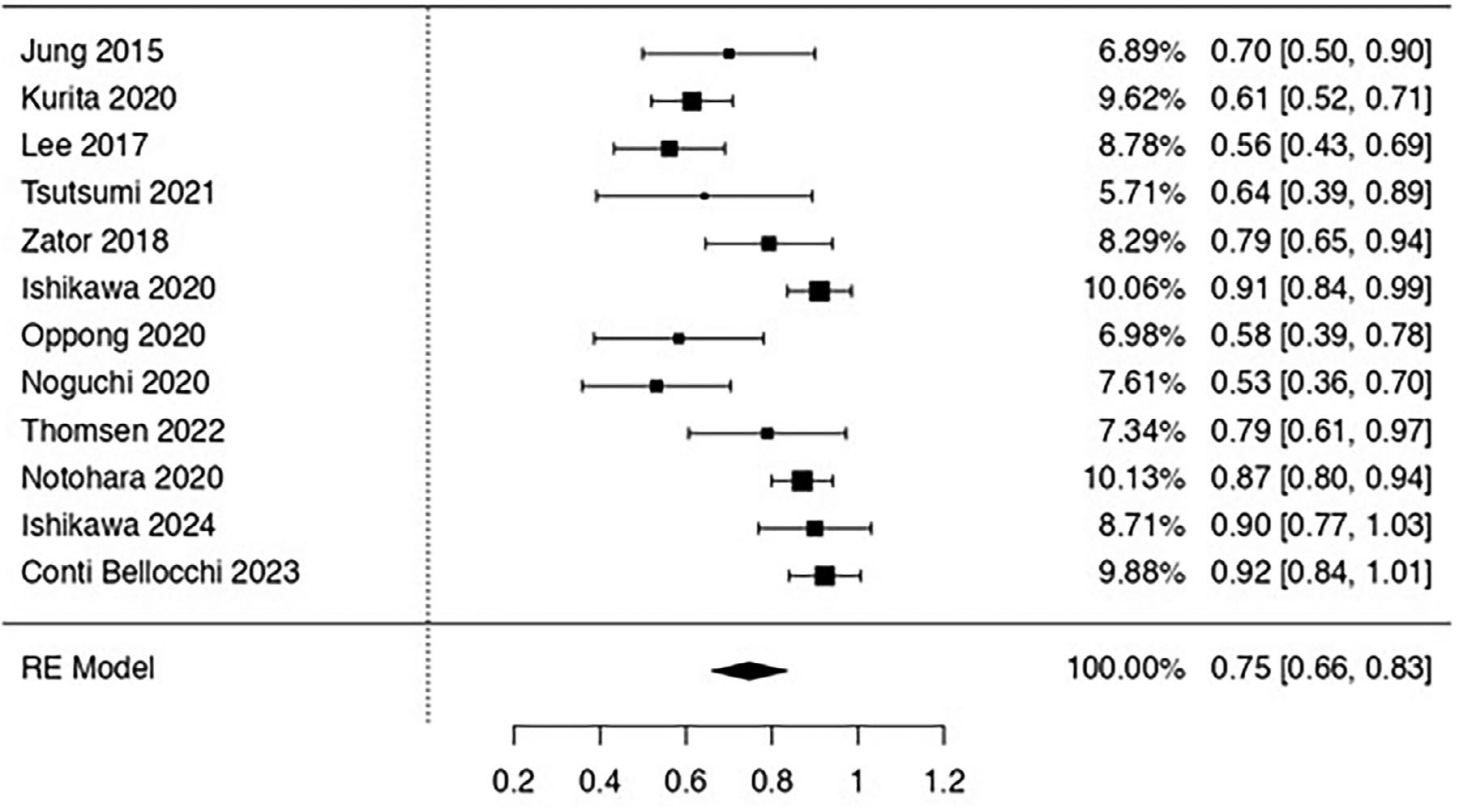
Pooled analysis assessing the diagnostic accuracy of endoscopic ultrasound‐guided tissue acquisition using fine‐needle biopsy in patients with autoimmune pancreatitis. Overall diagnostic accuracy was 75% (66%–83%; I^2^ = 83.5%).

As reported in Figure 2 and Table 2, the overall diagnostic accuracy of EUS‐TA using FNB was 75% (95%CI, 66%‐83%; I^2^ 83.5%). Subgroup analysis performed according to the kind of needle used showed a significant superiority of end‐cutting FNB over reverse bevel FNB (80%, 70%–90% vs. 49%, 21%–67%; *p* < 0.001). Of note, as reported in Table 2, a decrease in the heterogeneity was observed in the sensitivity analysis according to the FNB needle used (I^2^ 35.5% and 29%, respectively).

**TABLE 2:** Overall and subgroup analysis of main diagnostic outcomes. Subgroup analysis was performed based on the needle used. Numbers in parentheses indicate 95% confidence intervals.

Subgroup		No. of cohorts	No. of patients	Summary estimate (95% CI)	Within‐group heterogeneity (I^2^)
**Diagnostic accuracy**					
**Overall**		12	496	75% (66%–83%)	83.5%
**End‐cutting FNB needle**		8	370	80% (70%–90%)	35.5%
**Reverse bevel FNB needle**		4	97	49% (21%–67%)	29%
**End‐cutting needle**	**Franseen**	4	130	81% (68%–93%)	16.3%
	**Fork‐tip**	3	69	86% (74%–98%)	17.3%
	**Forward bevel**	3	121	67% (36%–97%)	92.3%
**Geographic location**	**East**	8	385	73% (61%–84%)	46.5%
	**West**	4	111	76.4% (65%–82%)	31.4%
**Autoimmune pancreatitis type**	**Type 1**	6	284	71% (56%–86%)	43.5%
**ICDC histological level of diagnosis**					
**Level 1**	**Overall**	10	377	47% (38%–57%)	74.16%
	**End‐cutting**	9	340	48% (37%–59%)	49.2%
**Level 2**	**Overall**	9	348	23% (16%–30%)	66.38%
	**End‐cutting**	8	330	23% (16%–30%)	50%

Abbreviations: CI, confidence interval; FNB, fine‐needle biopsy.

Further sub‐analysis performed according to the end‐cutting needle used showed higher accuracy with Franseen (81%, 68%‐93%) and Fork‐tip needle (86%, 74%–98%; *p* = 0.45), whereas a lower performance was observed with 20G forward bevel needle (67%, 36%–97%) although this result should be interpreted with caution due to the low number of studies in this specific subgroup.

The geographic location of the studies was also responsible for the high heterogeneity observed (I^2^ 46.5% and 31.4% in Eastern and Western studies, respectively) without a significant difference in terms of overall accuracy.

Finally, the aforementioned results were confirmed also when the analysis was restricted to AIP‐1 patients (71%, 56%–86%; I^2^ 43.5%).

No evidence of publication bias was observed in the accuracy analysis, as depicted in the funnel plot in Figure S1.

### 3.3 Secondary Outcomes

**FIGURE 3:**
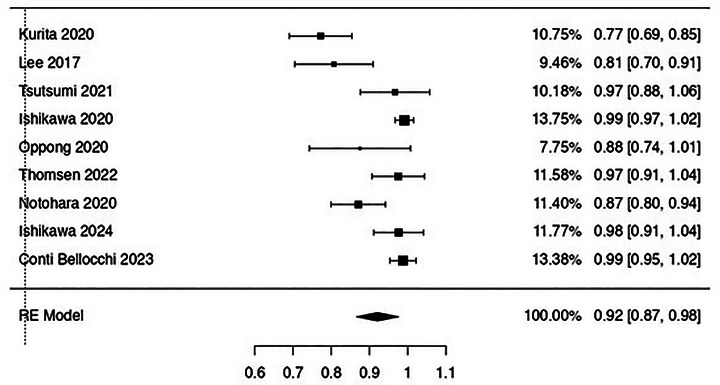
Pooled analysis assessing rates of sample adequacy achieved with endoscopic ultrasound‐guided tissue acquisition using fine‐needle biopsy. Overall sample adequacy was 92% (87%–98%; I^2^ = 87.2%).

Sample adequacy rate was 92% (87%–98%) with high evidence of heterogeneity (I^2^ 87.2%; Figure 3).

EUS‐TA using FNB provided level 1 of histological diagnosis according to ICDC criteria in 47% of cases (38%–57%; I^2^ 74.2%) and specifically in 48% of cases (37%–59%; I^2^ 49.2%) with end‐cutting needles (Table 2). An additional 23% (16%–30%) of patients obtained level 2 of ICDC histological diagnosis with FNB, whereof 23% (16%–30%; I^2^ 50%) with end‐cutting FNB needles.

**FIGURE 4:**
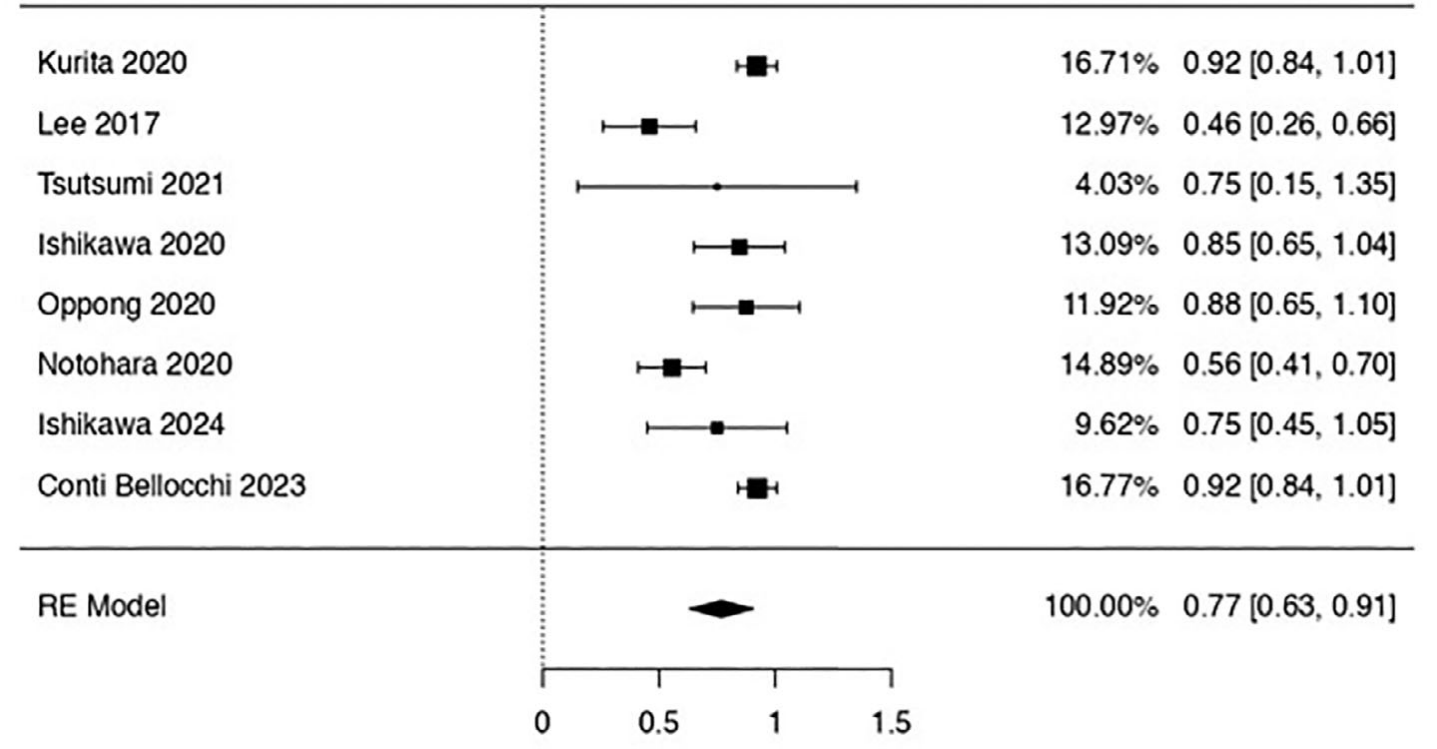
Pooled analysis assessing the rate of definitive histopathology of endoscopic ultrasound‐guided tissue acquisition using fine‐needle biopsy. Overall, the rate of definitive histopathology was 77% (63%–91%). Moderate evidence of heterogeneity was observed (I^2^ = 22.1%).

EUS‐TA using FNB provided a rate of definitive diagnosis as high as 77% (63%–91%), with moderate evidence of heterogeneity (I^2^ 22.1%; Figure 4).

As reported in Figure S2, the pooled rate of adverse events was 2% (1%–3%) with low evidence of heterogeneity (I^2^ 15%). Adverse events are detailed in Table S2 and were mainly mild episodes of self‐limiting pancreatitis after EUS‐TA using FNB.

## 4 Discussion

Preliminary results of EUS‐TA with newer end‐cutting FNB needles showed encouraging results that led to a conditional recommendation (in the absence of robust data from the literature) in favor of these needles in AIP patients in the recent European Society of Gastrointestinal Endoscopy (ESGE) guidelines [26]. With a meta‐analysis of 12 studies, we made several key observations. First, the overall diagnostic accuracy of EUS‐TA using FNB was 75%, and end‐cutting FNB showed a clear superiority over reverse bevel needles (80% vs. 49%). This result is significantly superior to the findings of our previous meta‐analysis, where diagnostic accuracy was only 54.7% overall and 63% with FNB [7]. When that meta‐analysis was published, only a few reports with end‐cutting needles were available, and most of the evidence was based on studies using the reverse bevel needle. These findings highlight the important impact of end‐cutting needles in the diagnostic algorithm of AIP patients.

On the other hand, rates of achievement of levels 1 and 2 of histological diagnosis according to ICDC criteria (47% and 23%, respectively) were only slightly superior to the results of our previous meta‐analysis (where level 1 and 2 was achieved in 44.2% and 21.9% of cases, respectively) [7]. Evidently, although the new device improved the accuracy, obtaining high‐quality core tissues for a complete histological diagnosis still represents a challenge for the endoscopist. The other two previous meta‐analyses [27, 28], including around 100 patients with EUS‐TA using FNB, showed higher diagnostic yield for level 1 or 2 histology criteria of AIP; however, both papers also included trucut core biopsies in the FNB group, which may have influenced the results significantly, and the limited number of patients did not allow to draw definitive conclusions.

Nevertheless, despite these undoubted improvements with newer FNB needles, the diagnostic accuracy in AIP is still suboptimal, particularly if compared to pancreatic adenocarcinoma patients. Obtaining adequate samples to meet the histologic ICDC criteria represents a real challenge for the endoscopist. These criteria are mainly based on findings described on surgical specimens that cannot be easily obtained in even a high‐quality biopsy sample. Therefore, in light of the outstanding histological performance of the newer generation FNB needles, our results should push the pathologists to reconsider the histological diagnostic criteria for AIP to define more feasible criteria based on FNB samples [29].

Moreover, most of the studies included patients with already a definitive AIP diagnosis based on radiological and serological criteria, where the utility of EUS‐TA using FNB is questionable and sometimes even misleading in the case of an inconclusive result.

To obviate this issue, we performed an analysis focused on the definitive histopathology, defined as the contribution of histologic findings obtained with EUS‐TA using FNB to the definitive diagnosis of AIP according to the ICDC, when the diagnosis was not possible clinically based on imaging, serology, or other organs' involvement. We found that EUS‐TA using FNB provided a 77% rate of definitive histopathology in this specific subset of patients, where EUS‐TA is really needed. Therefore, we can conclude that EUS‐TA using FNB may significantly impact the diagnosis of AIP in more than two‐thirds of patients.

No difference was observed between the two main end‐cutting needle devices, namely Franseen and Fork‐tip (81% and 86%, respectively), whereas a lower yield was found with 20G forward bevel needles (67%), but probably this result was due to the limited number of reports using this device, and it should be interpreted with caution.

High heterogeneity was registered in our analysis; however, multiple sensitivity analyses identified the kind of needle and the geographic location as the main sources of this heterogeneity, which, in fact, significantly decreased in the subgroup analysis based on these parameters. Other potential sources of heterogeneity could not be explored due to the lack of data, for example, the presence of a small proportion of AIP‐2 patients or the inclusion of patients with malignancy suspicion in some studies. Further data are needed to address this important point.

One of the pitfalls of the literature on AIP has always been that most available reports represent a regionalized experience deriving mainly from Japanese centers. In the last years, several Western series of EUS‐TA in these patients were published, and our meta‐analyses included four studies from Europe/USA [18, 20, 22, 25]; of note, sensitivity analysis based on geographic location of the studies did not show discordant results.

Most of the patients included in our meta‐analysis were diagnosed with AIP type 1; hence, our results should be considered applicable mainly to this subset of patients who represent the vast majority of AIP patients, particularly in Eastern countries, which largely contributed to the body of evidence on this topic.

Another key finding is the very high sample adequacy rate, which was beyond 90%, thus confirming the results of the literature in the field of EUS‐TA using FNB with end‐cutting needles [5, 30].

Finally, EUS‐TA using FNB is a safe technique with only a very limited number of patients experiencing mild adverse events, mainly mild pancreatitis, when used to evaluate AIP.

There are some limitations to our study. First, the limited number of comparative studies or RCTs did not allow a direct comparison between different FNB needles. Second, as mentioned above, the role of EUS‐TA using FNB specifically related to AIP‐2 could not be addressed due to the lack of available studies; therefore, our results should be considered applicable mainly to AIP‐1 patients. Third, being a benign condition, only a small proportion of patients enrolled in the included studies underwent surgery, thus meaning that the gold standard for accuracy analysis consisted mainly of the confirmation of diagnosis based on the clinical course during the follow‐up or the response to a steroid trial. However, all the included studies used the ICDC criteria for the diagnosis, supporting the robustness of our analysis. Finally, a subgroup analysis based on the needle size was not feasible due to the lack of data. However, several meta‐analyses demonstrated that both 22 and 25G FNB needles are equally effective in EUS‐TA for pancreatic masses [31]; therefore, the needle size is unlikely to influence the final outcomes.

In conclusion, our meta‐analysis demonstrates that the development of end‐cutting needles significantly improved the diagnostic performance of EUS‐TA using FNB in AIP patients. However, the diagnostic accuracy remains suboptimal compared to other conditions, such as pancreatic cancer. However, EUS‐TA using FNB may represent a valuable option for providing a definitive diagnosis in patients who cannot be diagnosed solely based on serology or imaging. The procurement of core tissues adequate for ICDC level 1 and 2 diagnosis remains relatively low, probably due to the strict histological requirements needed to fulfill these criteria.

## Author Contributions

**Antonio Facciorusso**: conceptualization, data extraction, studies assessment, and drafting manuscript; **Maria Cristina Conti Bellocchi**: literature search and revision for important intellectual content; **Nicolò De Pretis**: literature search and revision for important intellectual content; **Luca Frulloni**: revision for important intellectual content; **Stefano Francesco Crinò**: conceptualization, data extraction, studies assessment, and revision for important intellectual content.

## Conflicts of Interest

Stefano Francesco Crinò is a consultant for Oncosil Medical and AlphaTau and a paid speaker for Boston Scientific. The other authors declare no conflicts of interest.

## Funding

The authors received no specific funding related to this work.

## Ethics Statement

**Approval of the research protocol by an Institutional Reviewer Board**: N/A.

## Consent

N/A.

## Clinical Trial Registration

N/A.

## Supporting information




**TABLE S1**: Risk of bias assessment and quality of included studies.
**TABLE S2**: Adverse events reported in the included studies.
**FIGURE S1**: Funnel plot for diagnostic accuracy.
**FIGURE S2**: Forest plot for adverse event rate.
