# Supplementary material for: Endoscopic Ultrasound‐guided Fine‐Needle Biopsy With End‐Cutting Needles in Autoimmune Pancreatitis: A Systematic Review and Meta‐Analysis
Source: DEN Open. 2025 Nov 4;6(1):e70239. doi: 10.1002/deo2.70239 (PMC12584877; doi:10.1002/deo2.70239)
Supplement: Supplementary file 1 — TABLE S1: Risk of bias assessment and quality of included studies. TABLE S2: Adverse events reported in the included studies. FIGURE S1: Funnel plot for diagnostic accuracy. FIGURE S2: Forest plot for adverse event rate. [file DEO2-6-e70239-s001.docx]

**ONLINE SUPPLEMENT**

**Supplementary Table 2.** Adverse events reported in the included studies

| **Study, Year** | **Adverse events** |
| --- | --- |
| Jung 2015 | Mild pancreatitis (2 patients) |
| Kurita 2020 | Mild pancreatitis (1 patient)  Mild bleeding (1 patient) |
| Tsutsumi 2021 | 2 mild pancreatitis |
| Zator 2018 | Mild pain (3 patients)  Acute pancreatitis (2 patients) |
| Ishikawa 2020 | 2 mild pain |
| Noguchi 2020 | 2 mild pain  2 pancreatic fistula |
| Ishikawa 2024 | 1 mild pancreatitis |
| Conti Bellocchi 2023 | 2 mild pancreatitis |

**Supplementary Figure 1.** Funnel plot for diagnostic accuracy

**
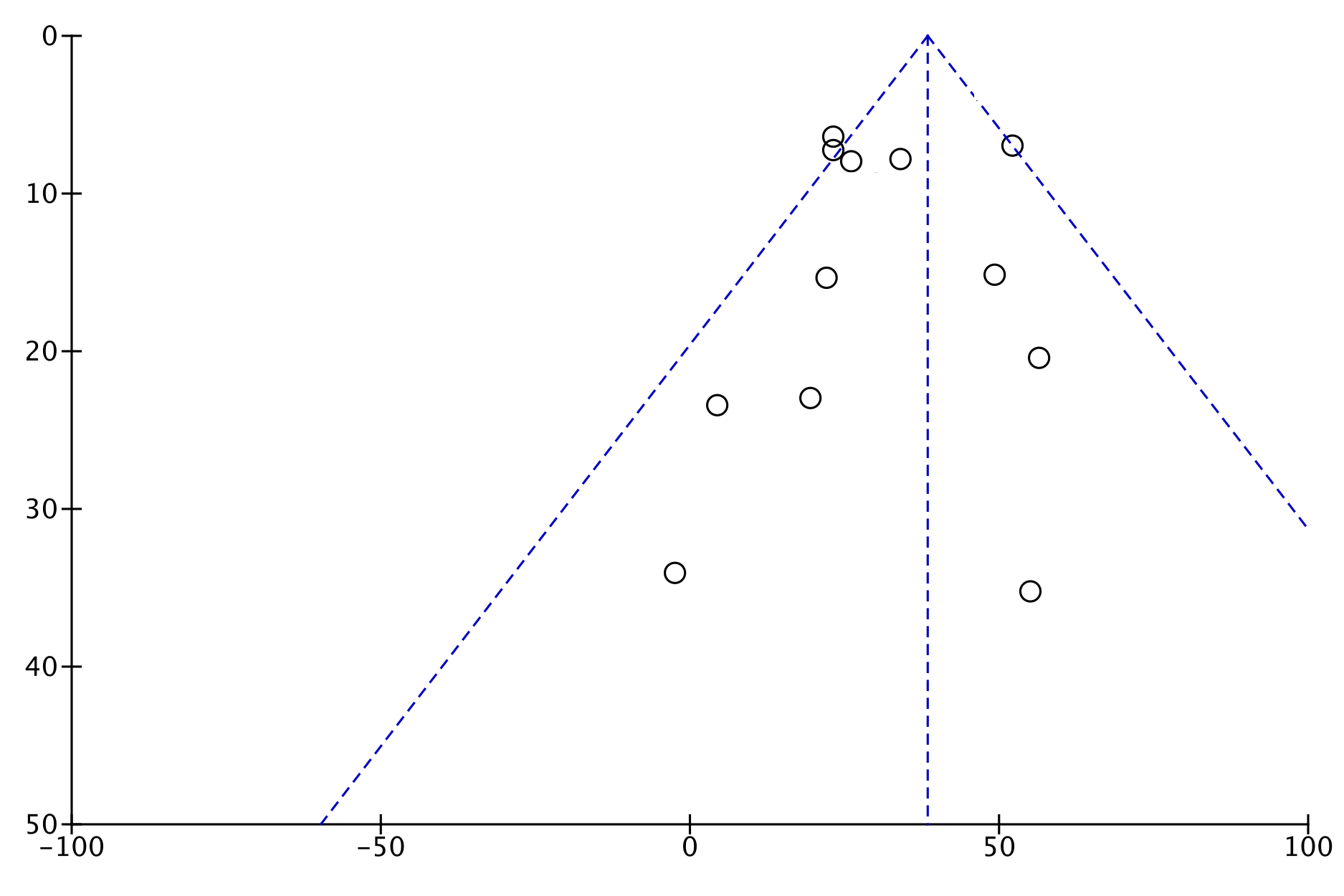
**

**Supplementary Figure 2.** Forest plot for adverse event rate

**
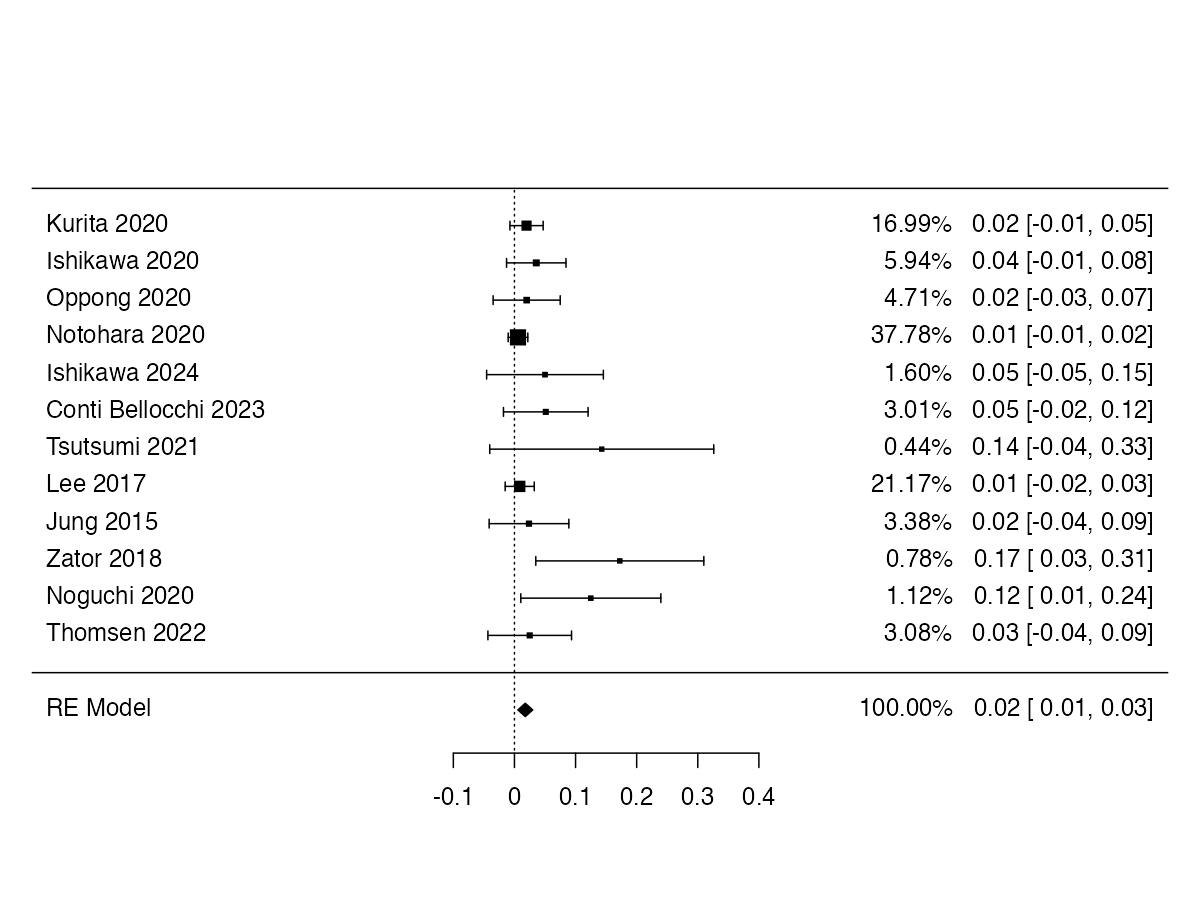
**
